# Predicting higher child BMI z-score and obesity incidence in Malaysia: a longitudinal analysis of a dynamic cohort study

**DOI:** 10.1186/s12889-024-18917-9

**Published:** 2024-05-27

**Authors:** Ruth Salway, Miranda Armstrong, Jeevitha Mariapun, Daniel D Reidpath, Sophia Brady, Mohamed Shajahan Yasin, Tin Tin Su, Laura Johnson

**Affiliations:** 1 Centre for Exercise, Nutrition & Health Sciences, School for Policy Studies, University of Bristol, 8 Priory Road, Bristol, BS8 1TZ UK; 2 Population Health Sciences, Bristol Medical School, University of Bristol, Canynge Hall, Bristol, BS8 2PN UK; 3 Clinical School Johor Bahru, Jeffrey Cheah School of Medicine and Health Sciences, Monash University Malaysia, Subang Jaya, Malaysia; 4 Institute for Global Health and Development, Queen Margaret University, Edinburgh, EH21 6UU Scotland; 5 South East Asia Community Observatory (SEACO), and Global Public Health, Jeffrey Cheah School of Medicine and Health Sciences, Monash University Malaysia, Subang Jaya, Malaysia

**Keywords:** Children, Adolescents, BMI, Intergenerational obesity, Cardiometabolic risk factors, Malaysia

## Abstract

**Background:**

To target public health obesity prevention, we need to predict who might become obese i.e. predictors of increasing Body Mass Index (BMI) or obesity incidence. Predictors of incidence may be distinct from more well-studied predictors of prevalence, therefore we explored parent, child and sociodemographic predictors of child/adolescent BMI z-score and obesity incidence over 5 years in Malaysia.

**Methods:**

The South East Asia Community Observatory in Segamat, Malaysia, provided longitudinal data on children and their parents (*n* = 1767). Children were aged 6–14 years at baseline (2013-14) and followed up 5 years later. Linear multilevel models estimated associations with child BMI z-score at follow-up, adjusting for baseline BMI z-score and potential confounders. Predictors included parent cardiometabolic health (overweight/obesity, central obesity, hypertension, hyperglycaemia), and socio-demographics (ethnicity, employment, education). Logistic multilevel models explored predictors of obesity incidence.

**Results:**

Higher baseline BMI z-score predicted higher follow-up BMI z-score both in childhood to late adolescence (0.60; 95% CI: 0.55, 0.65) and early to late adolescence (0.76; 95% CI: 0.70, 0.82). There was inconsistent evidence of association between child BMI z-score at follow-up with parent cardiometabolic risk factors independent of baseline child BMI z-score. For example, maternal obesity, but not overweight, predicted a higher BMI z-score in childhood to early adolescence (overweight: 0.16; 95% CI: -0.03, 0.36, obesity: 0.41; 95% CI: 0.20, 0.61), and paternal overweight, but not obesity, predicted a higher BMI z-score in early to late adolescence (overweight: 0.22; 95% CI: 0.01, 0.43, obesity: 0.16; 95% CI: -0.10, 0.41). Parental obesity consistently predicted five-year obesity incidence in early to late adolescence, but not childhood to early adolescence. An adolescent without obesity at baseline with parents with obesity, had 3–4 times greater odds of developing obesity during follow-up (incidence OR = 3.38 (95% CI: 1.14–9.98, mother) and OR = 4.37 (95% CI 1.34–14.27, father) respectively).

**Conclusions:**

Having a higher BMI z-score at baseline was a stronger predictor of a higher BMI z-score at follow-up than any parental or sociodemographic factor. Targeting prevention efforts based on parent or sociodemographic factors is unwarranted but early childhood remains a key period for universal obesity prevention.

**Supplementary Information:**

The online version contains supplementary material available at 10.1186/s12889-024-18917-9.

## Background

Obesity is a major public health concern, which increases the risk of developing non-communicable diseases (NCDs) such as diabetes, stroke and cardiovascular disease [1]. Children and adolescents with obesity are five times more likely to become adults with obesity, with approximately 80% of adolescents with obesity remaining so in adulthood [2]. Obesity in childhood and adolescence is independently associated with the development of NCDs later in life [3]. To date, no childhood obesity treatments show long-term success [4, 5], so preventing new incidence of obesity in childhood and adolescence is vital for long-term NCD prevention. Prevention is particularly important in low-and-middle-income countries (LMICs) where the prevalence of NCDs is lower but increasing rapidly [6]. In Malaysia, NCDs are the most common cause of death [7], and obesity prevalence in children and adolescents has more than doubled between 2011 and 2019 (6% and 15% respectively) [8, 9], pointing to a need to understand new incidence of obesity. Obesity rates differ by ethnicity, with higher rates among Malay and Indian and lower among Chinese ethnicities [9, 10]. Increasing obesity prevalence in Asian LMICs is generally attributed to changes in dietary and physical activity patterns caused by economic factors and urbanisation [11]. These include shifts towards more calorie-dense westernized foods and an increase in sedentary, indoor behaviours driven by a lack of open spaces or neighbourhood safety [12, 13]. Universal prevention efforts to improve eating and activity behaviours are generally ineffective, often because long-term behaviour change requires intensive and sustained interventions. In resource poor settings, targeting prevention strategies at populations subgroups most likely to develop obesity could be more cost-effective. Thus understanding predictors of obesity incidence as well as prevalence is essential to identifying such subgroups before they develop obesity.

Parent obesity is consistently associated with child obesity prevalence, but less is known about associations with incidence. Two meta-analyses estimated the odds of childhood obesity for a child with parents with overweight/obesity to be double that of a child with parents of healthy weight [14, 15]. Cross-sectional associations are also seen between parent and child body mass index (BMI), with stronger associations for those children with higher BMIs, suggesting an intergenerational transmission of risk for a high BMI [16, 17]. Maternal pre-pregnancy BMI is prospectively associated with offspring BMI in both childhood and adulthood [18], but it is not clear whether associations are gender-specific, with some studies showing a stronger maternal association [17, 19] while others show no differences between mothers and fathers [14, 20, 21]. Most studies of obesity prevalence are in high income countries, and systematic reviews suggest weaker associations in LMICs [14, 15]. In Malaysia, a previous cross-sectional analysis found a two-fold higher obesity prevalence among children with one or more parents with obesity or central obesity [22], and the prevalence of overweight mother-child pairs increased from 15 to 22% between 2006 and 2015 [23]. While prevalence studies identify groups in need of treatment, understanding predictors of greater BMI gain over time and obesity incidence could identify targets for prevention. Annual obesity incidence estimates decrease with age, from 3.2% in 5–13 year-olds to 1.8% in 13–18 year-olds in the US [24] with similar patterns in other high-income countries [25, 26]. However, obesity incidence is often lower in LMICs [27], and less is known about how incidence is related to parental risk factors in these countries. Identifying those children who may be at risk of developing obesity in future, based on current parental risk factors may allow intervention before obesity develops and thus reduce new incidence and future prevalence.

Few studies have explored the relationship between broader parental cardiometabolic risk factors, such as hypertension and hyperglycaemia, with child obesity although associations have been reported with parent cardiovascular health [28]and diabetes in parents [29, 30]. Intergenerational transmission of risk of NCDs could be via genetic or lifestyle mechanisms, and parents with hypertension or hyperglycaemia may have poorer diets and lower physical activity that may be shared with their children [31, 32]. To our knowledge, no studies have described differences in BMI or obesity incidence among children in Malaysia and little is known about the factors associated with higher gains in BMI and the development of obesity in this population. Therefore, we aimed to explore how parental weight status and cardiometabolic risk factors are longitudinally associated with child BMI z-score (follow-up adjusted for baseline) and obesity incidence.

## Methods

### Data

Analysis is reported following STROBE guidelines (Supplement). Data are from two health surveys from the South East Asia Community Observatory (SEACO) health and demographic surveillance system cohort in Malaysia [33], which undertakes annual enumeration of households within five of 11 sub-districts of the Segamat district. Population-wide individual level health surveys of participants aged 5 years and above were undertaken in 2013–14 and 2018-19, and collected questionnaire and biophysical measurement data on around 25,000 participants in each round (55–56% response rate of the total SEACO population), with around 10,000 participating at both timepoints.

**Fig. 1:**
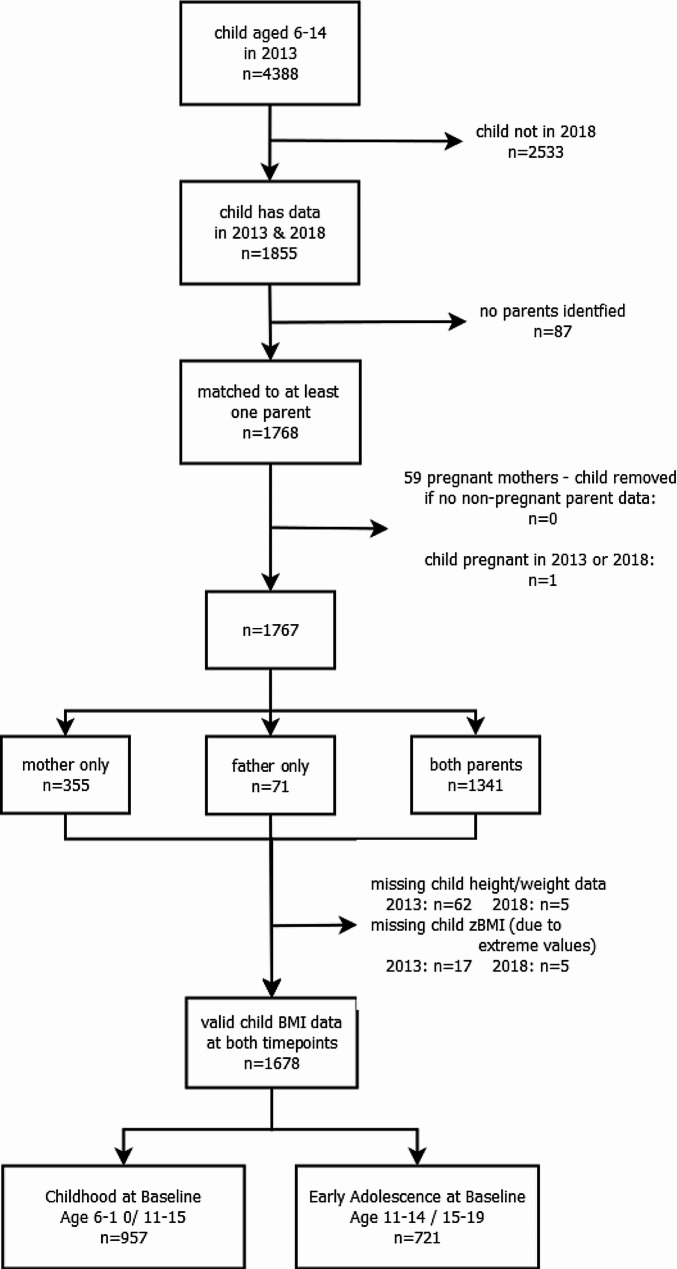
Flow diagram of participants

We analysed individual data on children aged 6–14 years at baseline (11–19 years at follow-up) and their parents from the SEACO health surveys, using household structure information taken from the enumeration in 2013-14 and 2018-19 to match parents and children [33]. Participants and households were linked across surveys using a unique SEACO participant ID. The analysis sample consisted of all children who had data at both timepoints and were matched to at least one parent with data. Fig. 1 shows the flow of participants from baseline to main analysis. We treated data for 59 mothers who were pregnant as missing and excluded one pregnant adolescent. Of the 4,388 children available at baseline, 1,855 (42%) had data at follow-up, with 1,768 (95%; 40% of the baseline cohort) matched to at least one parent, and 1,341 (72%; 31% of the baseline) having two parents identified. The final analysis sample size after multiple imputation (see below) was 1,768 (40% of baseline cohort).

#### Ethical considerations

Ethics approval for both surveys were obtained through the Monash University Human Research Ethics Committee: MUHREC (3837) for the Health Round Survey 2013 and MUHREC (13,242) for the Health Round Survey 2018. All participants gave informed consent which allows for secondary analysis without additional consent, and data was provided in anonymised form. As part of the SEACO Health Round surveys, adult participants received free health screenings (blood pressure, blood glucose, BMI and waist circumference), with referral letters provided to high-risk respondents for future health check-ups at local clinics.

#### Child BMI

Height and weight were measured without footwear or head gear (except a light scarf or veil) using a Transtek digital weighing scale with height gauge, (model GBS-721) by trained data collectors, with one measurement taken following the SEACO Standard Operating Protocol (SOP). Child BMI was calculated from weight and height and converted to age-adjusted standardised z-scores (BMI z-score) using the sex-specific World Health Organization (WHO) 2007 BMI reference for children aged 5–19. Children were classified with thinness, overweight or obesity according to WHO definitions [34] if the standardised BMI z-score was <-2 standard deviations (SD), >1SD and > 2SD, respectively, with all remaining children classified as healthy weight.

#### Parent risk factors

Anthropometric measurements of all participating parents (mothers and fathers) were taken by trained data collectors in the participant’s home, with parent height and weight measured in the same way as for children, following the SEACO SOP. Waist circumference was measured using an AccuFitness Myotape, with the measurement taken at the midpoint between the lower margin of the tip of the rib and the upper point of the iliac crest (hip bone), following the WHO STEPS protocol [35]. Blood pressure was recorded after participants had been sitting for at least 15 min. Three blood pressure measurements were taken using an Omron automated blood pressure monitor (HEM-7203) with 30–60 s between subsequent measurements, and the mean of the final two readings used, according to the SEACO SOP. Participants were asked how long since they had taken food or drink (other than water), and random non-fasting blood glucose was measured using a finger-prick Omron blood glucose monitoring system, (HGM-111). BMI (kg/m^2^) was calculated from height and weight and classified with thinness (< 18 kg/m^2^), overweight (> 25 kg/m^2^ and < 30 kg/m^2^), obesity (≥ 30 kg/m^2^), and of healthy weight otherwise [36], in line with previous analyses of the SEACO data [22]. Central obesity was defined using International Diabetes Federation (IDF) recommendations for Asian populations [37], as waist circumference ≥ 90 cm in men and ≥ 80 cm in women. Hypertension was defined as either systolic blood pressure ≥ 140 mmHg or diastolic blood pressure ≥ 90 mmHg [38]. Hyperglycaemia was defined as random non-fasting blood glucose ≥ 11.1 mmol/l [39].

#### Confounders

Parent and child age, gender and ethnicity were self-reported in the main SEACO census. Inconsistencies between the two timepoints were checked using date of birth and date of data collection with precedence given to age at baseline in 2013. We grouped children into two age groups, corresponding to Malaysian primary and secondary school ages: childhood at baseline (aged 6–10 years at baseline, aged 11–15 at follow-up) and adolescence at baseline (aged 11–14 years at baseline, aged 15–19 at follow-up)). Ethnicity was recoded into four groups: Malay, Chinese, Indian and Other (comprising indigenous (Orang Asli) and Other, grouped due to low numbers). Missing child ethnicity was derived from parent ethnicity, with preference given to 2013 data. Parental working status was classified as Working (part-time, full-time, casual) or Not Working (unemployed, housewife, student, retired). Highest parental education (up to primary, secondary, tertiary) was recorded for each parent.

### Statistical analysis

Baseline sample characteristics (child: age, gender, ethnicity, BMI z-score and weight category, and parent: age, gender, highest education, employment status, weight category, central obesity, hypertension and hyperglycaemia) were described using means and SDs, or percentages, as appropriate, by child gender and age group (results provided in main document). Child and Head of Household characteristics were compared for baseline and analysis samples, and for children with zero, one and two identified parents (see Appendix). Five-year incidence rate of obesity was calculated as the percentage without obesity at baseline with obesity at follow-up. Similarly, five-year remission rates were calculated as the percentage of those with obesity who were without obesity at follow-up, and five-year persistence rate as the percentage who remained with obesity (main document).

To investigate associations between parental risk factors (overweight/obesity, central obesity, hypertension and hyperglycaemia) and change in child BMI z-score, we modelled child BMI z-score in 2018 adjusted for baseline [40]. While concerns have been raised around the use of BMI z-scores for assessing change in child BMI for children with extreme BMIs, in particular those with severe obesity [41], this study focuses on estimating associations for the general population where BMI z-score has been found to be suitable for assessing change [42, 43] and so distortion in the extremes will have less of an impact, although associations will be underestimated for children with very high BMI. We fitted a linear multilevel model for children nested within parents, with child BMI z-score at follow-up as the outcome, adjusted for time to follow-up and baseline child BMI z-score; this assumes a linear change between baseline and follow-up. We first fitted models for each parental risk factor separately. We then fitted adjusted models, controlling for child characteristics (gender, age, ethnicity) and parent sociodemographic characteristics (age, working status and education Associations with parent hypertension and hyperglycaemia models were additionally adjusted for parent BMI category. The adjusted estimates are illustrated in Fig. 3 in the main document; estimates for unadjusted and adjusted models are given in the Appendix. We also fit models for the association between follow-up BMI z-score adjusted for baseline BMI z-score, and sociodemographic variables, specifically ethnicity, parental age, education and employment status. In a post-hoc analysis, we investigated associations between parental risk factors and obesity incidence, which focuses on children with higher BMI moving to a weight status of higher risk, and does not suffer from the same issues as using BMI z-score. We used logistic multilevel models, with a binary outcome for child with obesity at follow-up, restricted to those who were without obesity at baseline, and adjusted for time to follow-up, child characteristics, parent sociodemographic characteristics, and other parental risk factors as described above (main document). All analyses were stratified by age group, with separate models for mothers and fathers. A test for gender interactions did not support the need for stratification by gender, therefore results are reported for the whole sample combined. Adjusted models excluded child ethnicity ’Other’ due to small numbers. Descriptive summaries were performed in Stata v17 [44] and multilevel models were run in MLwiN v3.05 via the Stata command runmlwin, using restrictive iterative generalised least squares. Sensitivity analyses compared adjusted model estimates using imputed data with complete case analyses, and we repeated the analysis, first using Asian population BMI cut-offs for parental overweight (> 23 kg/m^2^ and < 25 kg/m^2^) and obesity (> 25 kg/m^2^) and second excluding children with thinness, as for these children an increase in BMI might be considered beneficial thus potentially violating the linearity assumption (Appendix).

#### Missing data

We restricted analysis to children with BMI data at both timepoints who could be matched to at least one parent (*n* = 1678; 38% of baseline). Missing data on gender, age and ethnicity for children and parents was taken from the other timepoint where available. Multiple imputation was used for parent risk factors to maximise the information used in the study and increase the precision of estimates [45]. We imputed missing parent data if they were included for at least one timepoint; note we did not impute child data at missing timepoints or BMI z-score.

Missing data was imputed using the jomo package in R (v4.0.2) via a simulation-based approach with a multivariate normal model to account for the multilevel structure. All analysis variables were included, and variables associated with missingness: total size of household, number of children and household structure (children/ parents, children/grandparents, and multi-generational/complex households). Continuous parental risk factors were imputed on a log scale, and parent BMI, central obesity, hypertension and hyperglycaemia were calculated from these. Twenty imputation datasets were created for each age group separately, using a burn-in of 500 iterations and 500 iterations between imputation datasets, selected by visual inspection. Model estimates were combined across the imputation datasets using Rubin’s rules [46].

## Results

There were 1,678 children included at both timepoints, with valid child BMI data, were not pregnant and were matched to at least one parent (Fig. 1). At baseline, 957 were aged 6–10 years (childhood at baseline) and 721 aged 11–14 (adolescence at baseline). Of these, 68% were Malay, 16% Chinese, 13% Indian and 2% Other, and 56% were female (Table 1). At baseline, 16% had obesity, 16% overweight and 16% thinness, with similar prevalences at follow-up. Compared to those excluded owing to missing child BMI data at a single timepoint, the children in the longitudinal analysis sample were a year younger, had lower baseline BMI z-score, were less likely to be male or Chinese, and came from households where the Head of Household had higher education and was less likely to be Indian (Table S1). Children with matched parents had higher BMI z-scores, were more likely to be male and were less likely to be Chinese (Table S2). Missing data varied between 8 and 27% for maternal and 20–36% for paternal variables (Table S3), primarily due to missing hypertension and hyperglycaemia. We imputed data on 9% of mothers and 24% of fathers; baseline characteristics were broadly similar between collected data and imputed data (Table S3). The average age of mothers was 41 years, with father slightly older at 45 years. Most parents (70%) were educated to secondary level, with most fathers in employment (91%) while mothers less likely to be employed (31%).

**Table 1:** Baseline child characteristics of the analysis sample by child gender and age group

		All	Gender		Age group^a^	
			Male	Female	Childhood at Baseline	Adolescence at Baseline
		*N* = 1678	*N* = 789	*N* = 889	*N* = 957	*N* = 721
% Female		53%			50%	56%
**Ethnicity**						
	Malay	68%	68%	68%	67%	67%
	Chinese	16%	15%	17%	17%	17%
	Indian	13%	14%	12%	12%	13%
	Aborigine	2%	2%	3%	3%	2%
	Other	< 0.5%	< 0.5%	< 0.5%	< 0.5%	< 0.5%
Age at baseline (2013)		11.2 (3.6)	11.2 (3.7)	11.4 (3.5)	8.0 (1.4)	12.4 (1.1)
Age at follow-up (2018)		16.1 (3.7)	16.0 (3.8)	16.2 (3.6)	12.8 (1.5)	17.3 (1.2)
BMI z-score at baseline		0.36 (1.51)	0.39 (1.61)	0.34 (1.41)	0.31 (1.58)	0.54 (1.39)
BMI z-score at follow-up		0.41 (1.51)	0.31 (1.59)	0.49 (1.40)	0.49 (1.50)	0.29 (1.48)
**BMI weight category**						
Baseline (2013)						
	Thinness	5%	6%	5%	7%	4%
	Healthy weight	61%	59%	62%	63%	59%
	Overweight	17%	16%	18%	15%	20%
	Obesity	17%	19%	15%	16%	18%
Follow-up (2018)						
	Thinness	5%	6%	3%	4%	5%
	Healthy weight	60%	59%	60%	57%	63%
	Overweight	18%	17%	19%	20%	16%
	Obesity	17%	17%	17%	18%	16%

^a^ Childhood group were aged 6–10 at baseline (11–15 at follow-up) and adolescence group aged 11–14 at baseline (16–19 at follow-up)

**Fig. 2:**
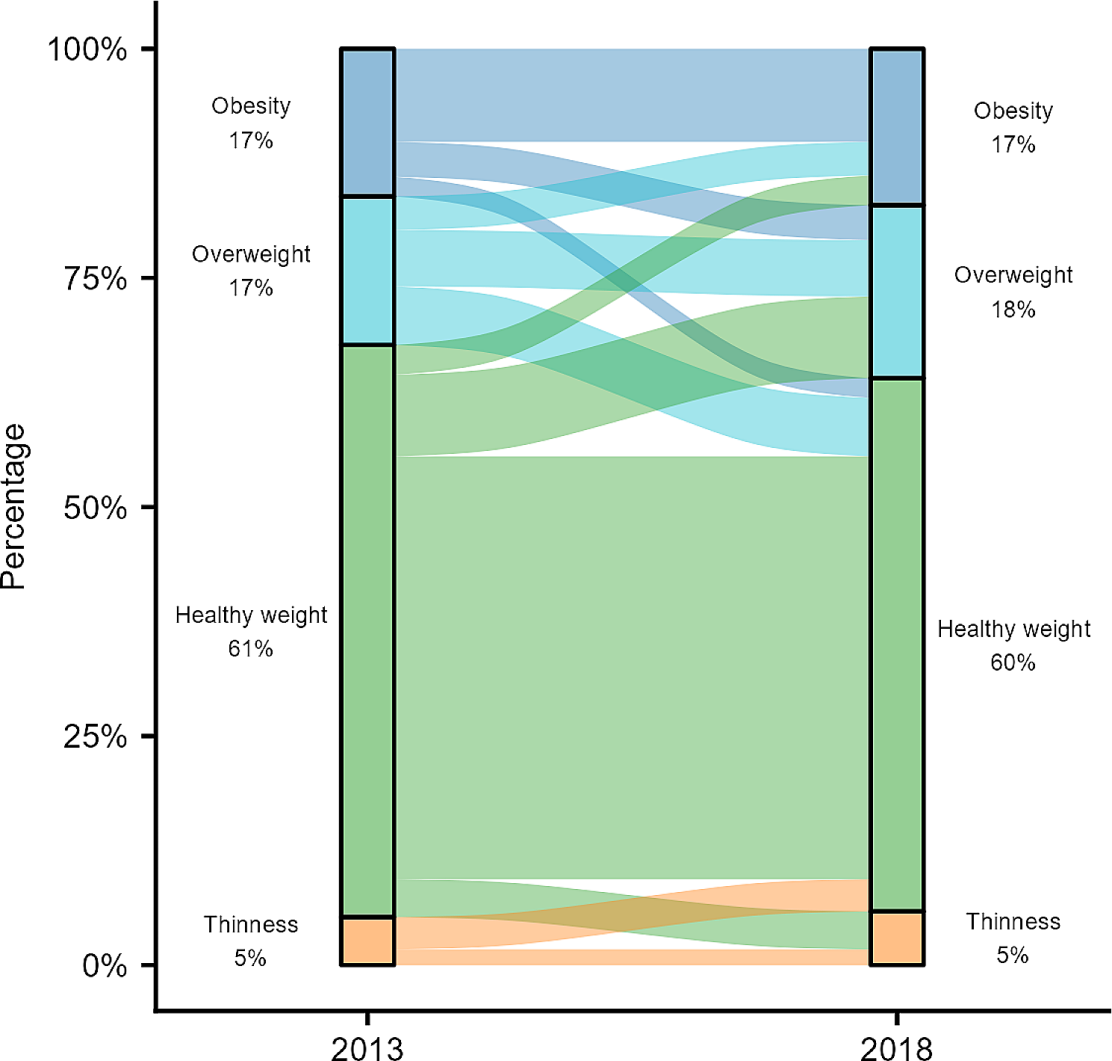
Change in child BMI category between 2013 and 2018

BMI z-score was higher at follow-up compared to baseline for girls, the childhood age-group at baseline. Whereas BMI z-score was lower at follow-up compared to baseline for the adolescent age-group at baseline (Table 1). Obesity prevalence was 16–18%, with movement between weight categories over time (Fig. 2). Five-year obesity incidence was higher in childhood (at baseline) than adolescence (at baseline) (10.8% and 6.1% respectively; Table 2). Five-year obesity remission rates of 42% were observed (Table 2). Of those participants with a healthy weight at baseline, 19% developed overweight or obesity at follow-up, compared to 7% developing thinness.

**Table 2:** Five-year incidence, remission and persistence rates

		All	Gender		Age group at baseline^a^	
			Male	Female	Childhood	Adolescence
Incidence^b^						
	Thinness	3.3%	4.3%	2.5%	2.4%	4.6%
	Overweight or obesity	17.5%	17.5%	17.5%	21.9%	11.1%
	Obesity	8.8%	8.1%	9.4%	10.8%	6.1%
Remission^c^						
	Thinness	73.9%	67.4%	80.4%	71.9%	78.6%
	Overweight or obesity	29.0%	33.1%	25.2%	32.6%	51.8%
	Obesity	41.7%	43.0%	40.3%	41.8%	41.5%
Persistence^d^						
	Thinness	26.1%	32.6%	19.6%	28.1%	21.4%
	Overweight or obesity	71.0%	66.9%	74.8%	67.4%	48.2%
	Obesity	58.3%	57.0%	59.7%	58.2%	58.5%

^a^ Childhood group were aged 6–10 at baseline (11–15 at follow-up) and adolescence group aged 11–14 at baseline (16–19 at follow-up)

^b^ Incidence: percentage of those not in BMI category at baseline, who are in category at follow-up

^c^ Remission: percentage of those in BMI category at baseline, who are not in category at follow-up

^d^ Persistence: percentage of those in BMI category at baseline, who are still in category at follow-up

**Fig. 3:**
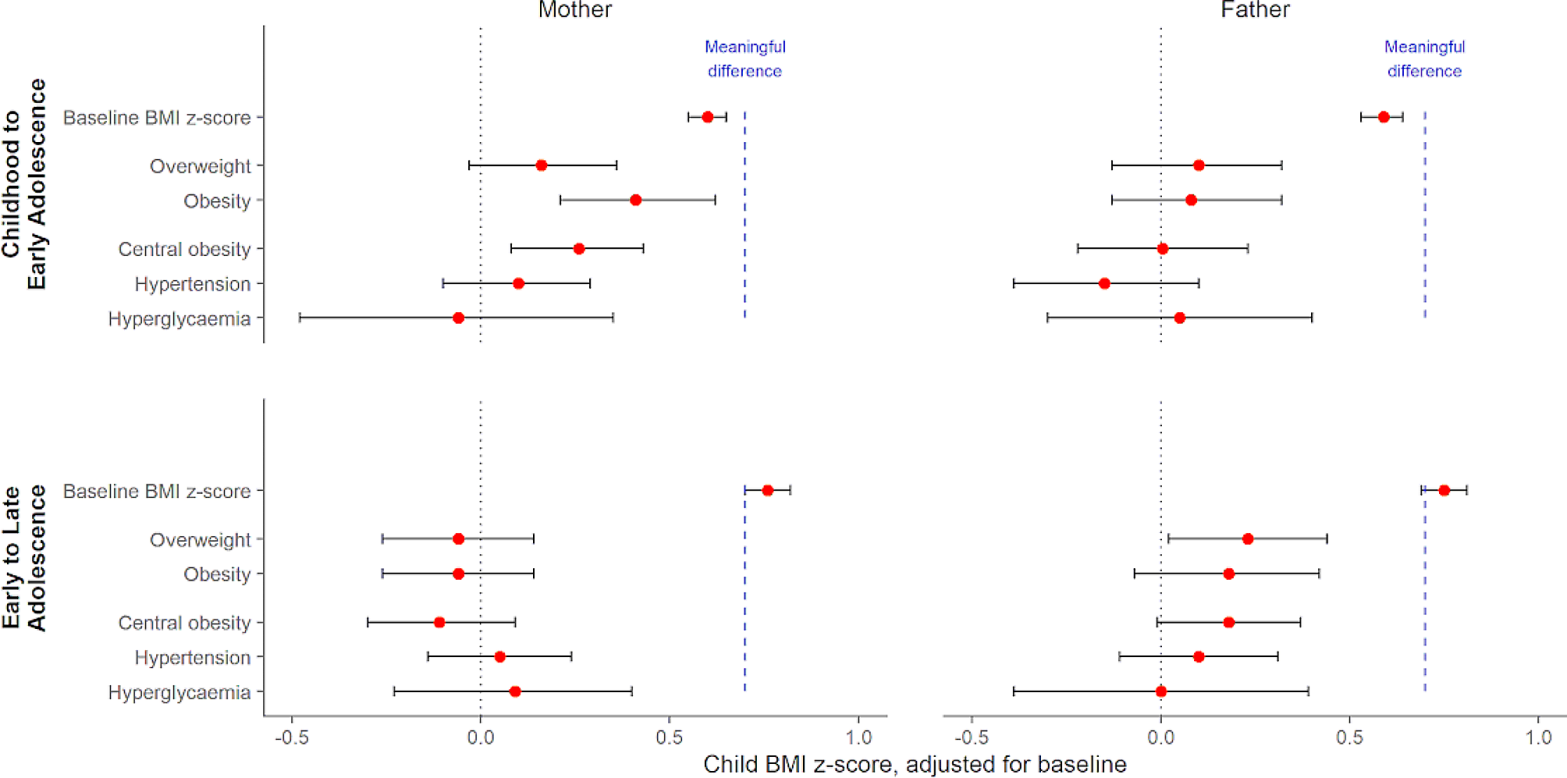
Association between parental risk factors and baseline-adjusted child BMI z-score at follow-up

Fig. 3 (and Table S4 & S5) shows modelled associations between parent cardiometabolic health and child BMI z-score at follow-up. For all models, higher child BMI z-score at baseline was the strongest predictor of a higher child BMI z-score at follow-up. Each unit of baseline BMI z-score was associated with a 0.60 (95% CI: 0.55, 0.65) higher follow-up BMI z-score for childhood to early adolescence and 0.76 (95% CI: 0.70, 0.82) higher for early to late adolescence. Associations with parental cardiometabolic risk factors were much smaller with weak or no evidence of association. In childhood to early adolescence, compared to healthy maternal weight, obesity (B = 0.41 (95% CI: 0.20, 0.61)), but not overweight (B = 0.16 (95% CI: -0.03, 0.36)) was associated with an increase in child BMI z-score of at follow-up (Table S4). While not associated with higher BMI z-score among younger participants (childhood to early adolescence), compared to a healthy paternal weight, overweight (B = 0.22 (95% CI: 0.01, 0.43)) but not obesity (B = 0.16 (95% CI: -0.10, 0.41)) showed small associations with a higher BMI z-score in older participants (early to late adolescence) (Table S4). Parental BMI and waist circumference were strongly correlated (0.73–0.75) and so associations with central obesity followed the same patterns as parental overweight/obesity. Associations between child BMI z-score at follow-up and baseline parent hypertension and hyperglycaemia (Table S4), ethnicity, parent employment status or educational attainment (Table S5) were small with weak or no evidence of association.

Model estimates and 95% confidence intervals by age strata and parent (see also Table S4). Models adjusted for baseline BMI z-score, time interval, child (gender, age, ethnicity) and relevant parent characteristics (age, education, other cardiometabolic risk factors). The dashed blue line represents the minimum change in BMI z-score for a meaningful clinical impact on child and adolescent lipids and blood pressure, based on a recent meta-analysis (El-Medany et al., 2020).

Childhood to early adolescence group were aged 6–10 at baseline (11–15 at follow-up) and early to late adolescence group aged 11–14 at baseline (16–19 at follow-up).

We saw similar patterns for obesity incidence, with stronger maternal associations in childhood to early adolescence, and paternal associations in early to late adolescence (Table 3). In childhood to early adolescence, the odds of obesity incidence were similar among those with a parent with overweight vs. a healthy weight parent (paternal OR = 0.67 (95% CI 0.33–1.37); maternal OR = 1.33 (95% CI: 0.71–2.48)). Obesity incidence odds were higher among the older age group, especially for those with a parent with obesity at 3–4 times higher (paternal OR = 4.37 (95% CI 1.34–14.27); maternal OR = 3.38 (95% CI: 1.14–9.98)). Associations with parental central obesity followed similar patterns, especially for maternal central obesity, where odds were higher than for BMI-based overweight/obesity (Table 3). There were no marked associations with parent hypertension or hyperglycaemia (ORs between 0.5 and 1.5 with wide confidence intervals).

**Table 3:** Odds ratios (OR) for association between parental factors at baseline and incidence of child obesity (imputed data)

		Childhood to early adolescence^a^		Early to late adolescence^a^	
		OR	95% CI	OR	95% CI
**Mother**			*N* = 749		*N* = 557
BMI category					
	Overweight^b^	1.33	(0.71, 2.48)	1.94	(0.66, 5.65)
	Obesity^b^	1.91	(0.98, 3.72)	3.65	(1.26, 10.54)
Central obesity^c^		2.44	(1.36, 4.41)	5.31	(1.53, 18.46)
Hypertension^c^		1.21	(0.69, 2.14)	1.52	(0.65, 3.54)
Hyperglycaemia^c^		0.80	(0.28, 2.23)	1.05	(0.24, 4.65)
**Father**			*N* = 622		*N* = 475
BMI category					
	Overweight^b^	0.68	(0.33, 1.39)	2.02	(0.65, 6.26)
	Obesity^b^	1.49	(0.78, 2.84)	3.88	(1.21, 12.48)
Central obesity^c^		1.68	(0.93, 3.04)	1.64	(0.59, 4.52)
Hypertension^c^		1.00	(0.52, 1.92)	0.51	(0.12, 2.14)
Hyperglycaemia^c^		1.17	(0.37, 3.63)	1.08	(0.10, 12.25)

Model adjusted for time interval, child (gender, age, ethnicity) and relevant parent characteristics (age, education, other cardiometabolic risk factors)

^a^ Childhood to early adolescence group were aged 6–10 at baseline (11–15 at follow-up) and early to late adolescence group aged 11–14 at baseline (16–19 at follow-up)

^b^ Compared to reference category ‘Thinness/Healthy weight’

^c^ Compared to reference category ‘Parent does not have specified risk factor’

Complete case analysis showed similar associations (Table S6), with slightly stronger associations for mothers with overweight. Sensitivity analyses were run for the association between child BMI z-score at follow-up and parental weight category using the overweight/obesity definition for Asian populations, which has lower BMI thresholds (Table S7). We saw similar patterns of associations to before, but with parental obesity rather than parental overweight; specifically associations with maternal obesity for those in childhood to early adolescence, and associations with paternal obesity for those in early to late adolescence. As the linearity assumption between baseline and follow-up BMI z-score did not hold for low values of baseline BMI z-score (Figure S1)., we repeated the analysis excluding children with thinness (Table S8) but found no difference in the reported associations between child BMI z-score at follow-up and parental cardiometabolic risk factors.

## Discussion

We have reported how parental cardiometabolic factors (overweight, obesity, central obesity, hypertension and hyperglycaemia) are associated with child BMI z-score at five-year follow-up, and the development of obesity in Malaysian children. Five-year increases in BMI z-score depended on child age at baseline, with larger increases between childhood and early adolescence compared to early and late adolescence. Previous cross-sectional analyses highlight associations between parental weight status and sociodemographic factors and child BMI z-score at a single timepoint [21, 22]. We found parental obesity was weakly associated with child BMI z-score differences, below values considered clinically meaningful, but associated with high odds of developing obesity, suggesting that targeted childhood obesity prevention strategies may need to focus on children of parents with obesity who may not be immediately at risk. For example, existing adult weight management programmes, such as ‘My body is fit and fabulous’ in Malaysia aimed at housewives [47], could also include advice and support aimed at the whole family to target children in childhood and early adolescence before they develop obesity.

In Segamat, obesity prevalence was estimated at 16% in 6–10 year olds, 18% in 10–14 year olds and 16% in those aged 15 or more, with overweight prevalence at 15%, 20% and 15% respectively. This is consistent with overweight and obesity prevalences of 15% across ages 5–18 reported for Malaysian children in the National Health and Morbidity Survey 2019 and elsewhere [9, 21, 23], but highlights variation with age, with higher rates during the early adolescent period. While obesity prevalence was relatively stable, this masked substantial change. Over five years, new obesity incidence was 8.8%, while 42% of those with obesity at baseline were without obesity at follow-up. Both the incidence and remission rates are slightly lower than those reported in high-income countries [24, 25] and we did not observe higher remission rates among younger children as other studies have reported [48–50]. In general, the majority of children who shift between BMI categories tend to do so at the boundaries. While some of the remission may be attributable to measurement error or regression to the mean, it may also be due to individual behaviour change, timing of maturation [48], or lower odds of remission associated with low birthweight [50].

Malaysia has a double burden of both thinness and overweight/obesity but we found more children with healthy weight at baseline developing overweight/obesity than thinness at follow-up (19% and 7% respectively). Our findings may reflect differences in LMICs where obesity rates are still increasing, with higher incidence in childhood not balanced by higher remission rates, leading to an increasing obesity prevalence over time, although conclusions are limited by having only two time points.

In childhood to early adolescence, maternal overweight/obesity were associated with higher mean BMI z-score at follow-up compared to mothers of healthy weight, whilst in early to late adolescence the associations of higher offspring BMI z-score were strongest with paternal overweight and obesity. Note that we have used WHO international BMI thresholds for parental overweight/obesity, but we found similar maternal and paternal patterns by child age group when using lower risk thresholds suggested for Asian populations, with the key difference being associations with parental obesity but not overweight, reflecting the shift in the thresholds. We observed similar patterns for central obesity, which is in line with the strong correlation between BMI and waist circumference. A recent meta-analysis [51] indicates a minimum mean increase in BMI z-score for a clinical impact on lipid profiles and blood pressure in 4–19 year olds is 0.7 z-scores. The associations we observed are over five years, and estimates (and confidence interval bounds) are much smaller than 0.7 and thus do not suggest associations are substantial enough to alter metabolic health in a clinically-relevant way. While larger cross-sectional associations between parental overweight/obesity and children’s BMI z-score [14, 15, 22] may be due to genetic predisposition to obesity, common obesogenic lifestyles, including diet and physical activity, due to shared living environment and/or behavioural factors passed down from parent to child [14], our analysis suggests these factors have a far smaller impact on the five-year difference in BMI z-score. We note, however, that our results may underestimate the association for those children with very high BMIs, and so further research on associations for children with severe obesity may be warranted.

There were no marked associations with parental hypertension, hyperglycaemia, or socioeconomic status, thus our study suggests that childhood obesity prevention strategies may be best targeted at those who have parents with overweight or obesity. The strongest associations observed were with BMI z-score at baseline, as children with higher initial BMI experienced larger five-year increases than expected compared to WHO references.

While increasing BMI z-scores can indicate growth in excess of normal expectations for a given age and sex, higher-than-average BMI is not in itself a cause for concern if children remain on the same trajectories into adolescence, because they will stay within the healthy BMI range. Both BMI z-score and overweight/obesity definitions in children are based on comparison to a historic cross-sectional reference population (in this study, the WHO reference population) and thus differences we observe may be a result of different underlying characteristics of populations, such as the timing of pubertal growth spurts, rather than necessarily cause for concern. Figure S2 (see Appendix) shows that median BMI was higher than the WHO growth reference at all ages for boys and girls, with the rate of change in BMI temporally shifted, so the fastest rate of increase occurred 1–2 years younger. The strong associations with baseline BMI z-score suggest that excessive growth trajectories are initiated earlier in childhood and are perpetuated into adolescence [52–54]. Thus, the small associations between parental risk factors and offspring BMI z-score may be because those children predisposed to obesity, having already lived with overweight or obesity from a young age. However, these difficulties in assessing change in BMI in childhood make it challenging to interpret results.

Five-year obesity incidence was higher between childhood and early adolescence than between early and late adolescence (10.8% and 6.1% respectively), consistent with patterns elsewhere of higher incidence at younger ages [24, 25], and in line with the theory that excessive growth tends to occur earlier in childhood. Associations between parental overweight/obesity and child obesity incidence were larger in early to late adolescence than in childhood to early adolescence with parental overweight associated with a doubling in odds of five-year obesity incidence and parental obesity associated with a 3–4 times higher odds. Note that BMI changes considerably during puberty with a growth spurt and the adiposity rebound, which will occur predominantly within the childhood to early adolescence group, and may account for the weaker associations within this group. In contrast, the stronger associations in the early to late adolescence group, once puberty is more established, highlights that a child with one or both parents with obesity may not develop obesity until late adolescence, and so may not have been identified as at risk when younger. This is of concern because associations with obesity in adulthood are stronger for those with obesity in adolescence than in childhood [2], and thus indicate that this group of children may require targeting at a younger age even if they do not yet have obesity. Associations between parental obesity and adolescent obesity incidence but not BMI z-score may indicate a differential importance of parent weight for child BMI across the distribution of child BMI [16, 17]. For example, children with higher baseline BMI z-scores would need a smaller increase in BMI z-score (such as is associated with having a parent with obesity) to move into the overweight or obesity category. Thus, parental obesity may have a stronger association for children closer to the cut-off for obesity at baseline, especially in early adolescence.

We saw some evidence of different associations by parent gender, with maternal weight dominating in childhood to early adolescence and paternal weight in early to late adolescence. Maternal obesity before and during pregnancy (a key developmental period) is associated with offspring BMI in childhood, potentially driven by intrauterine, genetic or lifestyle factors such as smoking [53, 55]. Some cross-sectional studies have found stronger maternal associations [17, 19] and our results suggest that associations may continue into childhood, possibly reflecting the common role of the mother as primary caregiver in younger children, especially in more traditional communities, with more influence over lifestyle factors such as diet and physical activity. However, we found this was replaced by a weaker paternal association in early to late adolescence. Thus maternal associations in childhood may thus result in earlier puberty [56], and an earlier adolescent increase in BMI, while paternal weight is associated with higher BMI at the end of adolescence. However, all these associations were small and other evidence is inconclusive, especially for fathers. While much of the current evidence focuses on maternal associations, fathers are under-represented, and more research is needed to determine the paternal role throughout childhood and adolescence. Understanding further our findings of a stronger paternal association in early to late adolescence is of particular importance as this is a critical time for BMI trajectories into adulthood.

### Strengths and limitations

This study uses prospective data with a robust sampling design in the SEACO cohort, resulting in high levels of parental data, especially for fathers who are typically under-represented, with most studies focusing on mothers [23]. Parental cardiometabolic risk factors were measured objectively rather than using self-report measures. However, because data were collected at household level, matching children to parents was difficult especially with multiple families in a household and we were unable to distinguish between biological and non-biological parents. We also excluded a substantial number of children due to missing data at follow-up, and did not have data on lifestyle factors such as diet or physical activity. While multiple imputation maximises the available information in the data, this is under a missing at random assumption. Exploring changes in BMI in this age group is challenging due to puberty and childhood growth patterns, and so care should be taken in interpreting results. Furthermore, Segamat is a semi-rural region so is not generalisable to the wider Malaysian population, although overweight/obesity estimates are similar [23]. Finally, our analysis focuses on the general population; estimates of associations may be underestimated for those children with severe obesity.

## Conclusions

In this Malaysian cohort, child/adolescent obesity prevalence was stable at 16–18%, with a five-year incidence of obesity at 8.8%. Parental overweight/obesity was prospectively associated with slightly higher child BMI z-score after adjusting for baseline, but the largest follow-up BMI z-scores were among children with a higher baseline BMI z-score. These findings support the importance of childhood as a key period for obesity prevention, rather than later intervention based on parent cardiometabolic risk. However, those in early adolescence with higher BMI z-score and at least one parent with obesity may be at an increased risk of becoming obese during late adolescence.

### Electronic supplementary material

Below is the link to the electronic supplementary material.

Supplementary Material 1

## Data Availability

Data are from an ongoing prospective cohort study and are available from SEACO by completion of a data application form to: https://www.monash.edu.my/seaco/research-and-training/how-to-collaborate-with-seaco.

## Abbreviations

BMI: Body mass index
IDF: International Diabetes Federation
LMIC: Low-and-middle-income country
NCD: Non-communicable disease
OR: Odds ratio
SEACO: South East Asia Community Observatory
WHO: World Health Organization
