# Supplementary material for: Predicting higher child BMI z-score and obesity incidence in Malaysia: a longitudinal analysis of a dynamic cohort study
Source: BMC Public Health. 2024 May 27;24:1408. doi: 10.1186/s12889-024-18917-9 (PMC11129495; doi:10.1186/s12889-024-18917-9)

# Supporting Information

Predicting increases in child BMI z-score and obesity incidence in Malaysia: a longitudinal analysis of a dynamic cohort study

Ruth Salway^1,2^, Miranda Armstrong^1,3^, Jeevitha Mariapun^4^, Daniel D Reidpath^5^, Sophia Brady^1^, Mohamed Shajahan Yasin^4^, Tin Tin Su ^6*^, Laura Johnson^2*^

** These authors share the joint senior authorship.*

**Corresponding author:** Dr Ruth Salway, Population Health Sciences, Bristol Medical School, University of Bristol, Canynge Hall, Bristol BS8 2PN

Email: ruth.salway@bristol.ac.uk

**Contents**

Table S1: Comparison of characteristics of sample and children with only one time points

Table S2: Comparison of characteristics of children with zero, one and two parents matched

Table S3: Summary of missing data and comparison of complete case and imputed data

Table S4: Association between parental cardiometabolic factors at baseline and change in child BMI z-score

Table S5: Association between child and parental sociodemographic characteristics at baseline and change in child BMI z-score (imputed data)

Table S6: Association between parental cardiometabolic factors at baseline and change in child BMI z-score, adjusted for confounders (complete cases only)

Table S7: Association between parental BMI category (Asian population definitions) at baseline and child BMI z-score at follow-up, adjusted for confounders (complete cases only)

Table S8: Association between parental cardiometabolic factors at baseline and change in child BMI z-score, excluding underweight children

Figure S1: Association between baseline and follow-up BMI z-score with loess smoother

**Table S1: Comparison of characteristics of sample and children with only one time points**

|  | Age group at baseline | | |
| --- | --- | --- | --- |
|  | Aged 6-15 at baseline:  2013 data only^1^ | Aged 6-15 at baseline & 11-19 at follow-up:  2013 and 2018 data^1^ | Aged 11-19 at follow-up:  2018 data only^1^ |
| Total | 3113 | 1820 | 2357 |
| **CHILD CHARACTERISTICS** | | | |
| % female | 48% | 54% | 49% |
| Malay | 70% | 67% | 66% |
| Chinese | 20% | 17% | 24% |
| Indian | 8% | 12% | 6% |
| Orang Asli | 2% | 2% | 3% |
| **Baseline** | | | |
| Age: mean(SD) | 11.2 (3.0) | 10.0 (2.5) |  |
| BMI z-score: mean(SD) | 0.47 (1.48) | 0.38 (1.52) |  |
| Thinness | 15% | 17% |  |
| Healthy weight | 50% | 49% |  |
| Overweight | 19% | 16% |  |
| Obesity | 16% | 17% |  |
| **Follow-up** |  |  |  |
| Age: mean(SD) |  | 14.8 (2.5) | 14.8 (2.5) |
| BMI z-score: mean(SD) |  | 0.39 (1.50) | 0.40 (1.51) |
| Thinness |  | 19% | 18% |
| Healthy weight |  | 46% | 49% |
| Overweight |  | 18% | 16% |
| Obesity |  | 17% | 17% |
| **HEAD OF HOUSEHOLD CHARACTERISTICS: baseline** | | | |
| % up to primary education | 37% | 27% |  |
| % Secondary or higher | 63% | 73% |  |
| Malay | 68% | 72% |  |
| Chinese | 16% | 18% |  |
| Indian | 14% | 8% |  |
| Age: mean(SD) | 50.3 (11.5) | 49.1 (11.3) |  |
| **HEAD OF HOUSEHOLD CHARACTERISTICS: follow-up** | | | |
| % up to primary education |  | 37% | 37% |
| % Secondary or higher |  | 63% | 64% |
| % Malay |  | 71% | 69% |
| % Chinese |  | 15% | 22% |
| % Indian |  | 12% | 6% |
| Age: mean(SD) |  | 54.0 (11.4) | 53.2 (11.9) |

^1^ Comparison is between children who are eligible for inclusion in the analysis, that is aged 6-15 at baseline, which corresponds to aged 11-19 at follow-up.

**Table S2: Comparison of characteristics of children with zero, one and two parents matched**

|  | No parent data | One parent data | Two parents |
| --- | --- | --- | --- |
| N | 88 | 427 | 1340 |
| Age at baseline | 9.5 (32.6) | 9.8 (2.6) | 9.8 (2.5) |
| % female | 59% | 53% | 53% |
| Malay | 52% | 62% | 70% |
| Chinese | 30% | 21% | 16% |
| Indian | 14% | 13% | 12% |
| Orang Asli | 5% | 4% | 2% |
| **Baseline (2013)** |  |  |  |
| BMI z-score | 0.08 (1.55) | 0.42 (1.49) | 0.39 (1.51) |
| Thinness | 24% | 17% | 17% |
| Healthy weight | 52% | 49% | 50% |
| Overweight | 9% | 17% | 17% |
| Obesity | 15% | 17% | 17% |
| Household size: median (IQR) | 3 (2) | 4 (3) | 5 (2) |
| Number of children: median (IQR) | 2 (2) | 2 (1) | 2 (1) |
| **Follow-up (2018)** |  |  |  |
| BMI z-score | 0.11 (1.63) | 0.46 (1.53) | 0.40 (1.49) |
| Thinness | 31% | 18% | 18% |
| Healthy weight | 40% | 43% | 47% |
| Overweight | 11% | 20% | 18% |
| Obesity | 18% | 19% | 17% |
| Household size: median (IQR) | 4 (2) | 4 (2) | 5 (2) |
| Number of children: median (IQR) | 2 (1) | 2 (2) | 2 (2) |

**Table S3: Summary of missing data and comparison of complete case and imputed data**

|  |  | | Missing | | Complete case | Imputed |
| --- | --- | --- | --- | --- | --- | --- |
|  |  | | N | % | Mean (sd) or % | Mean (sd) or % |
| **Child** | | |  | |  |  |
| % Female | | | 0 | 0% | 53% | 53% |
| Age at baseline | | | 0 | 0% | 9.9 (2.5) | 11.3 (3.6) |
| Age at follow-up | | | 0 | 0% | 14.7 (2.6) |  |
| Ethnicity | | | 0 | 0% |  |  |
|  | Malay | |  |  | 67% | 68% |
|  | Chinese | |  |  | 17% | 16% |
|  | Indian | |  |  | 13% | 13% |
|  | Other | |  |  | 3% | 3% |
| BMI z-score at baseline (2013) | | | 0 | 0% | 0.41 (1.51) | 0.36 (1.51) |
| BMI z-score at follow-up (2018) | | | 0 | 0% | 0.41 (1.50) | 0.41 (1.50) |
| Change in BMI z-score | | | 0 | 0% | 0.02 (1.24) | 0.02 (1.24) |
| **Mother** | | 0 | | 4% |  |  |
| Age | | | 68 | 4% | 40.6 (7.4) | 40.6 (7.4) |
| Highest education: | | | 138 | 8% |  |  |
|  | Primary | |  |  | 21% | 21% |
|  | Secondary | |  |  | 71% | 71% |
|  | Tertiary | |  |  | 8% | 8% |
| BMI | | | 140 | 8% | 27.7 (6.2) | 28.5 (16.9) |
|  | Up to healthy weight | |  |  | 37% | 37% |
|  | Overweight | |  |  | 33% | 32% |
|  | Obesity | |  |  | 30% | 30% |
| Central obesity | | | 174 | 10% | 68% | 67% |
| Hypertension | | | 459 | 27% | 21% | 32% |
| Hyperglycaemia | | | 459 | 27% | 7% | 9% |
| **Father** | | | 335 | 20% |  |  |
| Age | | | 335 | 20% | 45.0 (8.5) | 45.0 (8.5) |
| Highest education: | | | 507 | 30% |  |  |
|  | Primary | |  |  | 23% | 23% |
|  | Secondary | |  |  | 70% | 69% |
|  | Tertiary | |  |  | 7% | 7% |
| BMI | | | 515 | 31% | 26.7 (5.6) | 26.7 (9.5) |
|  | Up to healthy weight | |  |  | 43% | 45% |
|  | Overweight | |  |  | 36% | 32% |
|  | Obesity | |  |  | 21% | 22% |
| Central obesity | | | 501 | 30% | 44% | 45% |
| Hypertension | | | 606 | 36% | 25% | 28% |
| Hyperglycaemia | | | 606 | 36% | 8% | 11% |

**Table S4: Association between parental cardiometabolic factors at baseline and child BMI z-score at follow-up (imputed data)**

|  |  | Age group at baseline^1^ | | | | | | | |
| --- | --- | --- | --- | --- | --- | --- | --- | --- | --- |
|  |  | **Childhood** | | | | **Adolescence** | | | |
|  |  | Model 1 | | Model 2 | | Model 1 | | Model 2 | |
|  |  | Change | 95% CI | Change | 95% CI | Change | 95% CI | Change | 95% CI |
| **Mother** | |  |  |  | **N=892** |  |  |  | **N=680** |
| BMI category^2^ | |  |  |  |  |  |  |  |  |
|  | Overweight | 0.16 | (-0.03, 0.36) | 0.16 | (-0.03, 0.36) | -0.03 | (-0.23, 0.17) | -0.06 | (-0.26, 0.14) |
|  | Obesity | 0.41 | (0.21, 0.61) | 0.41 | (0.20, 0.61) | 0.11 | (-0.11, 0.32) | 0.04 | (-0.17, 0.26) |
| Central obesity^3^ | | 0.24 | (0.06, 0.41) | 0.25 | (0.07, 0.43) | -0.03 | (-0.21, 0.16) | -0.12 | (-0.31, 0.07) |
| Hypertension^3^ | | 0.12 | (-0.09, 0.32) | 0.10 | (-0.10, 0.29) | 0.05 | (-0.15, 0.24) | 0.05 | (-0.14, 0.24) |
| Hyperglycaemia^3^ | | 0.02 | (-0.38, 0.43) | -0.06 | (-0.48, 0.35) | 0.13 | (-0.22, 0.48) | 0.09 | (-0.23, 0.40) |
| **Father** | |  |  |  | **N=742** |  |  |  | **N=572** |
| BMI category^2^ | |  |  |  |  |  |  |  |  |
|  | Overweight | 0.09 | (-0.13, 0.32) | 0.11 | (-0.12, 0.34) | 0.25 | (0.04, 0.47) | 0.22 | (0.01, 0.43) |
|  | Obesity | 0.10 | (-0.15, 0.34) | 0.10 | (-0.15, 0.35) | 0.25 | (-0.01, 0.50) | 0.16 | (-0.10, 0.41) |
| Central obesity^3^ | | 0.02 | (-0.19, 0.23) | 0.02 | (-0.22, 0.26) | 0.21 | (0.03, 0.40) | 0.17 | (-0.02, 0.36) |
| Hypertension^3^ | | -0.17 | (-0.40, 0.06) | -0.15 | (-0.39, 0.10) | 0.12 | (-0.10, 0.33) | 0.10 | (-0.11, 0.31) |
| Hyperglycaemia^3^ | | 0.04 | (-0.30, 0.38) | 0.05 | (-0.30, 0.40) | 0.09 | (-0.31, 0.48) | -0.001 | (-0.39, 0.39) |

Model 1 = model adjusted for baseline BMI z-score only

Model 2 = model adjusted additionally for child (gender, age, ethnicity) and the relevant parent characteristics (age, education, other CVD risk factors).

^1^ Childhood group were aged 6-10 at baseline (11-15 at follow-up) and adolescence group aged 11-14 at baseline (16-19 at follow-up).

^2^ Compared to reference category ‘Thinness /Healthy weight’

^3^ Compared to reference categories ‘Parent does not have specified risk factor’

**Table S5: Association between child and parental sociodemographic characteristics at baseline and child BMI z-score at follow-up (imputed data)**

|  |  | Age group at baseline^1^ | | | | | | |
| --- | --- | --- | --- | --- | --- | --- | --- | --- |
|  |  | Childhood | | | | Adolescence | | |
|  |  | Change | | 95% CI | | Change | 95% CI | |
| **Model for mother risk factors** | | |  | **N=892** | |  | **N=680** | |
| Child baseline z-score | | 0.60 | | (0.55, 0.65) | | 0.76 | (0.70, 0.82) | |
| Child gender (female) | | 0.16 | | (0.01, 0.30) | | 0.44 | (0.29, 0.60) | |
| Child age (per year) | | -0.13 | | (-0.18, -0.08) | | 0.12 | (0.04, 0.19) | |
| Child ethnicity | |  | |  | |  |  | |
| Chinese^2^ | | 0.07 | | (-0.15, 0.29) | | -0.12 | (-0.34, 0.10) | |
| Indian^2^ | | -0.002 | | (-0.25, 0.25) | | 0.20 | (-0.05, 0.46) | |
| Mother age (per 10 years) | | 0.03 | | (-0.09, 0.15) | | 0.09 | (-0.04, 0.22) | |
| Mother education | |  | |  | |  |  | |
| Secondary^3^ | | -0.07 | | (-0.29, 0.16) | | 0.05 | (-0.16, 0.27) | |
| Tertiary^3^ | | -0.03 | | (-0.37, 0.30) | | -0.03 | (-0.48, 0.34) | |
| Working^4^ | | -0.02 | | (-0.20, 0.16) | | 0.09 | (-0.09, 0.28) | |
| **Model for father risk factors** | | |  | | **N=742** |  | | **N=572** |
| Child baseline z-score | | 0.59 | | (0.53, 0.64) | | 0.75 | (0.68, 0.81) | |
| Child gender (female) | | 0.10 | | (-0.07, 0.26) | | 0.33 | (0.16, 0.50) | |
| Child age (per year) | | -0.14 | | (-0.20, -0.09) | | 0.10 | (0.03, 0.18) | |
| Child ethnicity | |  | |  | |  |  | |
| Chinese^2^ | | 0.03 | | (-0.22, 0.28) | | -0.18 | (-0.42, 0.07) | |
| Indian^2^ | | 0.10 | | (-0.18, 0.39) | | 0.13 | (-0.16, 0.42) | |
| Father age (per 10 years) | | 0.01 | | (-0.11, 0.12) | | -0.08 | (-0.20, 0.04) | |
| Father education | |  | |  | |  |  | |
| Secondary^3^ | | 0.14 | | (-0.12, 0.41) | | 0.05 | (-0.18, 0.29) | |
| Tertiary^3^ | | 0.32 | | (-0.14, 0.78) | | 0.09 | (-0.31, 0.48) | |
| Working^4^ | | -0.12 | | (-0.48, 0.25) | | -0.21 | (-0.55, 0.13) | |

Model adjusted for time interval, parent cardiometabolic risk factors (BMI category, hypertension and hyperglycaemia)

^1^ Childhood group were aged 6-10 at baseline (11-15 at follow-up) and adolescence group aged 11-14 at baseline (16-19 at follow-up).

^2^ Compared to reference category ‘Malay’

^3^ Compared to reference categories ‘Up to primary’

^4^ Compared to reference category ‘Not working’

**Table S6: Association between parental cardiometabolic factors at baseline and child BMI z-score at follow-up, adjusted for confounders (complete cases only)**

|  |  |  | | Age group at baseline^1^ | | | | | |
| --- | --- | --- | --- | --- | --- | --- | --- | --- | --- |
|  |  |  | | Childhood | | | Adolescence | | |
|  |  |  | | Est | | 95% CI | Est | 95% CI | |
| **Mother** | | |  | | **N=536** | |  | **N=533** |  |
|  | BMI category | | |  | |  |  |  | |
|  |  | Overweight^2^ | | 0.29 | | (0.04, 0.55) | -0.02 | (-0.23, 0.20) | |
|  |  | Obesity^2^ | | 0.42 | | (0.15, 0.69) | 0.11 | (-0.12, 0.33) | |
|  | Central obesity^3^ | | | 0.23 | | (-0.01, 0.46) | -0.15 | (-0.35, 0.05) | |
|  | Hypertension^3^ | | | 0.07 | | (-0.18, 0.32) | 0.11 | (-0.08, 0.30) | |
|  | Hyperglycaemia^3^ | | | 0.17 | | (-0.29, 0.62) | -0.15 | (-0.49, 0.20) | |
| **Father** | | |  | | **N=549** | |  | **N=474** |  |
|  | BMI category | | |  | |  |  |  | |
|  |  | Overweight^2^ | | 0.12 | | (-0.13, 0.36) | 0.26 | (0.04, 0.48) | |
|  |  | Obesity^2^ | | 0.09 | | (-0.19, 0.37) | 0.17 | (-0.11, 0.44) | |
|  | Central obesity^3^ | | | -0.01 | | (-0.24, 0.21) | 0.14 | (-0.07, 0.35) | |
|  | Hypertension^3^ | | | -0.20 | | (-0.46, 0.06) | 0.09 | (-0.16, 0.33) | |
|  | Hyperglycaemia^3^ | | | 0.09 | | (-0.19, 0.37) | -0.03 | (-0.42, 0.36) | |

Model adjusted for BMI z-score at baseline and all confounders

^1^ Childhood group were aged 6-10 at baseline (11-15 at follow-up) and adolescence group aged 11-14 at baseline (16-19 at follow-up).

^2^ Compared to reference category ‘Thinness /Healthy weight’

^3^ Compared to reference categories ‘Parent does not have specified risk factor’

**Table S7: Association between parental BMI category (Asian population definitions) at baseline and child BMI z-score at follow-up, adjusted for confounders (complete cases only)**

|  |  |  | | Age group at baseline^1^ | | | | | |
| --- | --- | --- | --- | --- | --- | --- | --- | --- | --- |
|  |  |  | | Childhood | | | Adolescence | | |
|  |  |  | | Est | | 95% CI | Est | 95% CI | |
| **Mother** | | |  | | **N=536** | |  | **N=533** |  |
|  |  | Overweight^2^ | | 0.23 | | (-0.11, 0.57) | 0.08 | (-0.22, 0.37) | |
|  |  | Obesity^2^ | | 0.43 | | (0.18, 0.69) | 0.08 | (-0.15, 0.32) | |
| **Father** | | |  | | **N=549** | |  | **N=474** |  |
|  |  | Overweight^2^ | | -0.09 | | (-0.41, 0.22) | 0.06 | (-0.25, 0.38) | |
|  |  | Obesity^2^ | | 0.06 | | (-0.21, 0.33) | 0.25 | (0.00, 0.50) | |

Model adjusted for BMI z-score at baseline and all confounders

^1^ Childhood group were aged 6-10 at baseline (11-15 at follow-up) and adolescence group aged 11-14 at baseline (16-19 at follow-up).

^2^ Compared to reference category ‘Thinness /Healthy weight’

**Table S8: Association between parental cardiometabolic factors at baseline and child BMI z-score at follow-up, excluding underweight children (imputed data)**

|  |  |  | | Age group at baseline^1^ | | | | |
| --- | --- | --- | --- | --- | --- | --- | --- | --- |
|  |  |  | | Childhood | | | Adolescence | |
|  |  |  | | Est | | 95% CI | Est | 95% CI |
| **Mother** | | |  | | **N=731** | |  | **N=547** |
|  | BMI category | | |  | |  |  |  |
|  |  | Overweight^2^ | | 0.06 | | (-0.11, 0.24) | -0.10 | (-0.30, 0.10) |
|  |  | Obesity^2^ | | 0.18 | | (-0.001, 0.36) | -0.01 | (-0.22, 0.20) |
|  | Central obesity^3^ | | | 0.13 | | (-0.03, 0.29) | -0.06 | (-0.25, 0.13) |
|  | Hypertension^3^ | | | 0.11 | | (-0.06, 0.28) | 0.02 | (-0.15, 0.20) |
|  | Hyperglycaemia^3^ | | | 0.06 | | (-0.28, 0.40) | -0.08 | (-0.36, 0.21) |
| **Father** | | |  | | **N=613** | |  | **N=464** |
|  | BMI category | | |  | |  |  |  |
|  |  | Overweight^2^ | | 0.06 | | (-0.14, 0.26) | 0.22 | (0.01, 0.42) |
|  |  | Obesity^2^ | | 0.12 | | (-0.10, 0.33) | 0.07 | (-0.17, 0.31) |
|  | Central obesity^3^ | | | 0.12 | | (-0.06, 0.32) | 0.13 | (-0.06, 0.32) |
|  | Hypertension^3^ | | | -0.18 | | (-0.38, 0.02) | 0.07 | (-0.15, 0.28) |
|  | Hyperglycaemia^3^ | | | -0.01 | | (-0.34, 0.33) | 0.05 | (-0.32, 0.42) |

Model adjusted for BMI z-score at baseline and all confounders

^1^ Childhood group were aged 6-10 at baseline (11-15 at follow-up) and adolescence group aged 11-14 at baseline (16-19 at follow-up).

^2^ Compared to reference category ‘Healthy weight’

^3^ Compared to reference categories ‘Parent does not have specified risk factor’

Figure S1: Association between baseline and follow-up BMI z-score with loess smoother


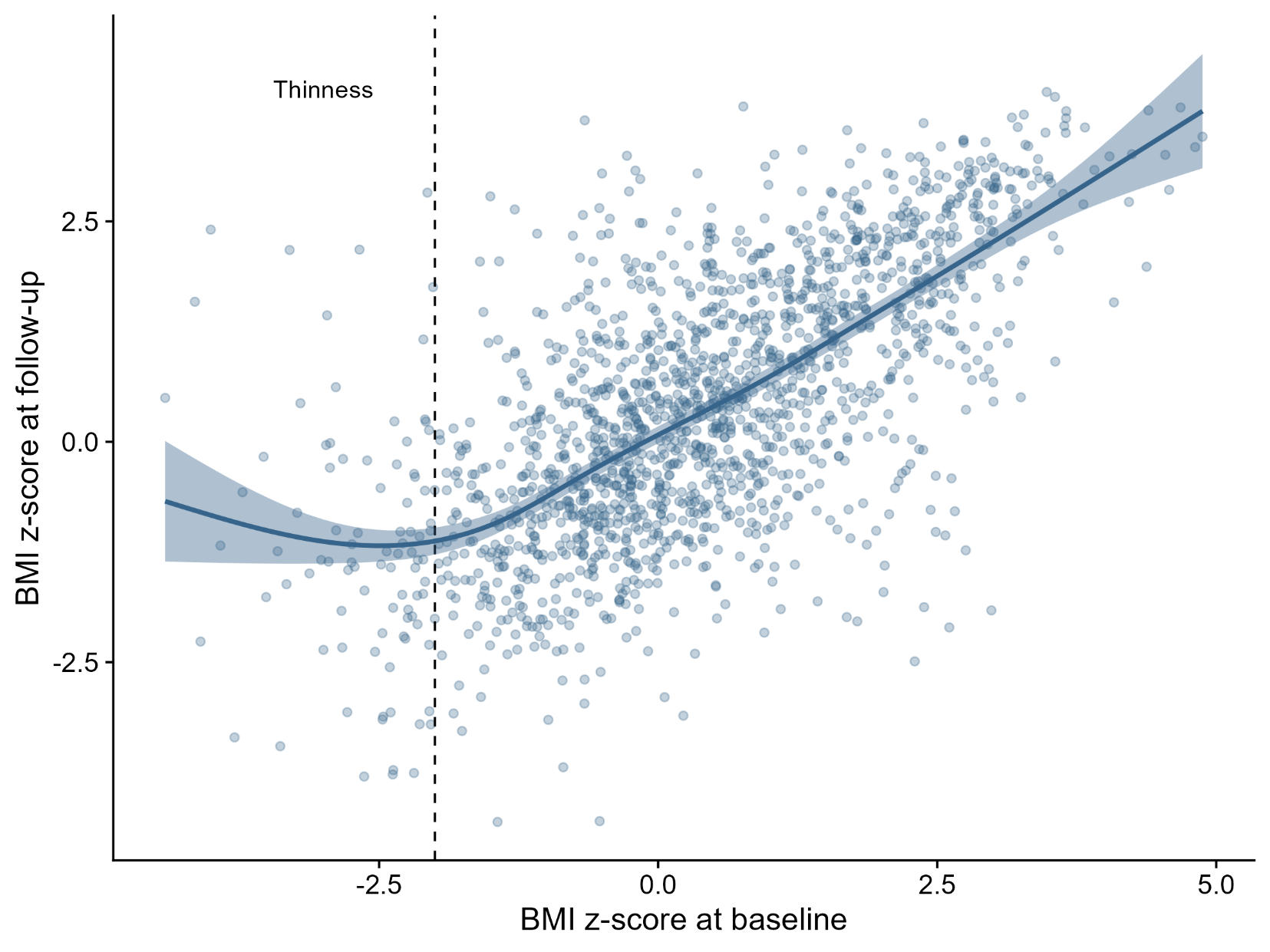

Supplement: Supplementary file 1 — Supplementary Material 1 [file 12889_2024_18917_MOESM1_ESM.docx]
